# Analytical Study of Stress Distributions around Screws in Flat Mandibular Bone under In-Plane Loading

**DOI:** 10.3390/bioengineering10070786

**Published:** 2023-06-30

**Authors:** Jinxing Huo, Jan-Michaél Hirsch, E. Kristofer Gamstedt

**Affiliations:** 1 Division of Applied Mechanics, Department of Materials Science and Engineering, Uppsala Univeristy, Box 35, SE-751 03 Uppsala, Sweden; jixning.huo@sandvik.com; 2 Department of Surgical Sciences, Oral & Maxillofacial Surgery, Uppsala University, SE-751 85 Uppsala, Sweden; jan.hirsch@regionstockholm.se

**Keywords:** bone fixation, orthotropic plate theory, analytical analysis, stress distribution

## Abstract

A known complication for mechanically loaded bone implants is the instability due to screw loosening, resulting in infection and the non-union of fractures. To investigate and eventually prevent such bone degradation, it is useful to know the stress state in the bone around the screw. Considering only in-plane loadings and simplifying the mandibular bone into an orthotropic laminated plate, the analysis was reduced to a two-dimensional pin-loaded plate problem. An analytic model, based on the complex stress analysis, was introduced to the bone biomechanics field to obtain the stress distributions around the screw hole in the bone. The dimensionless normalized stresses were found to be relatively insensitive to the locations of the screw hole over the mandible. Parametric analyses were carried out regarding the friction coefficient and load direction. It was found that the load direction had a negligible influence. On the contrary, the friction coefficient had a significant effect on the stress distributions. Whether the screw was well bonded or not thus played an important role. The proposed analytic model could potentially be used to study bone failure together with stress-based failure criteria.

## 1. Introduction

Fractures of human bones, e.g., the mandible, require adequate fixation in a correct occlusal position in order to allow the absorption of masticatory forces. Adopting plates fixed with screws is the standard procedure for the treatment of mandibular fractures currently, mainly because of its advantages of minimal tissue injury and high fixation stability [1]. However, bone loss is observed in patients after fracture treatment, especially when load bearing plates are used or bone grafting is involved. Resorption of bone surrounding the screws will consequently result in screw loosening, which in turn may cause instability, bone infection, further loosening, and occasionally osteomyelitis [2,3,4]. This leads to the need for surgical revision, removing the fixation material and new adequate fixation with plates and screws [5]. The failure is most often due to the incorrect analyses of surgeons about the biomechanical stability requirement in a specific fracture location and pattern and/or the wrong choice of material and an inadequate anatomical location for osteosynthesis not providing mechanical stability. From a mechanical perspective, the stress shielding and the high peak stresses at the interface are believed to be determinant for bone loss [6,7,8,9,10]. Thus, better understanding of stress distributions around screw holes is of great importance for preventing implant failures.

Several studies have been performed in the past few decades to investigate the biomechanics of bones around artificial implants. Most of the research is based on finite element simulations [11,12,13]. They provided detailed and precise stress distributions in the bone at the cost of a considerably large amount of time and computations. An analytic model to capture trends and parametric influences would be useful in the general understanding and for the design approach. The human mandible consists of two cortical layers and an intermediate layer of cancellous bone. For simplification, mandibular bones were idealized and treated as thin laminated plates in this work. This is acceptable since, in this work, only the in-plane loads were considered. The biomechanical problem could thus be converted into the well-solved pin-loaded plate problem, which has been researched substantially [14,15,16,17,18]. Among the theoretical investigations, most are analytical studies based on the complex stress function method [16]. De Jong adopted the general solution of the complex stress function to analyze stress distributions around a hole in a linear elastic plate loaded by a rigid pin [14]. The study was limited to frictionless and tight-fit connection. Hyer and Klang combined the complex stress function method with a collocation method in a general stress analysis, in which the deformation of the pin, clearance, and friction between the pin and the plate were all accounted for [17]. Zhang and Ueng obtained a compact analytic solution for the stress functions, which satisfies the major displacements on the hole loaded by a rigid, perfectly fitting pin [18].

The aim of the current work was to revive and introduce the complex stress analysis method into the bone biomechanics field for predicting the stress distribution around a screw-loaded hole in human mandibular bone. Parametric studies of the stress distribution in the bone regarding the relevant design parameters were easily conducted by utilizing an analytic model derived with the complex stress analysis method. Since the stresses determined were averaged with respect to the bone thickness, the stress distribution was a coarse approximation. Stress approximations in the cortical and cancellous layers can, however, be obtained from the elastic properties of these layers. The idea of the compact analytic model is to provide rough, but timely guidelines in the design process of the screws and the locations of fixation in the bones. For more accurate studies for cases where an in-plane pin loading and plate analogy cannot be assumed, an analytical approach is most likely not possible. In that case, one must resort to a full three-dimensional finite element analysis. Notably, such analytic models could easily be expanded and applied to other types of flat bone, such as the skull, in human bodies.

## 2. Stress Analysis

**Figure 1:**
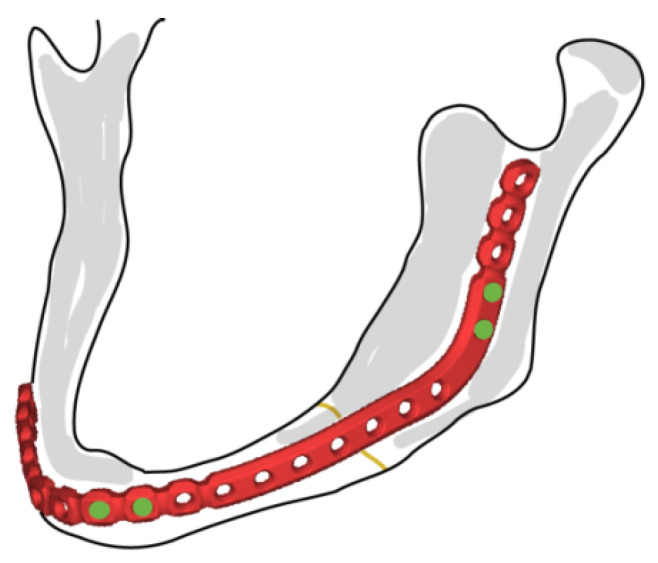
Commercial mandibular plate and fixation screws (DePuy Synthes).

Prior to the analytical analysis, the following simplifications were made and are listed regarding the screw and mandibular bone, schematically illustrated in Figure 1.

(1). The screws are supposed to be symmetrically loaded herein. As reported, through-thickness bicortical screw placement is often used for the rigid fixation of mandibular fractures [19]. Thus, an in-plane loaded rigid pin in a through hole was considered reasonable here. The effects of transverse pull-out loads and contributions from large moments deserve special attention and necessitate a full three-dimensional finite element study.
(2). The screw-load direction is arbitrary with respect to the principal material direction, as the loading direction has an influence on the strength of pin-loaded anisotropic plates [20,21].
(3). The screw was assumed to be infinitely rigid. According to Hyer et al. [22], the stiffness of the pin has a minor effect on stress distribution around the hole.
(4). Threads on the screw and screw hole were neglected.
(5). The location of the screw hole in the bone was assumed to be far away from the edge of the bone. The bone was considered to be of uniform thickness and flat in the vicinity of the screw.
(6). The contact between the screw and the bone was considered, and the friction coefficient was assumed to be constant over the contact region.

### 2.1. Elastic Properties of Human Bones

The elastic properties of bones are of fundamental interest in functional morphology, which describe how anatomical structures resist or deform by external loads [23]. In this context, the mandible was assumed to be homogenous and elastically orthotropic [24]. The elastic properties of mandibular bone have been experimentally determined [25,26,27], and the values for different regions of the mandibular cortical bone are presented in Table 1. The values were taken from [28], which was based on the experimental study in [29]. The material directions were in accordance with local Cartesian coordinate systems, in which Direction 3 is normal to the bone plane.

**Figure 2:**
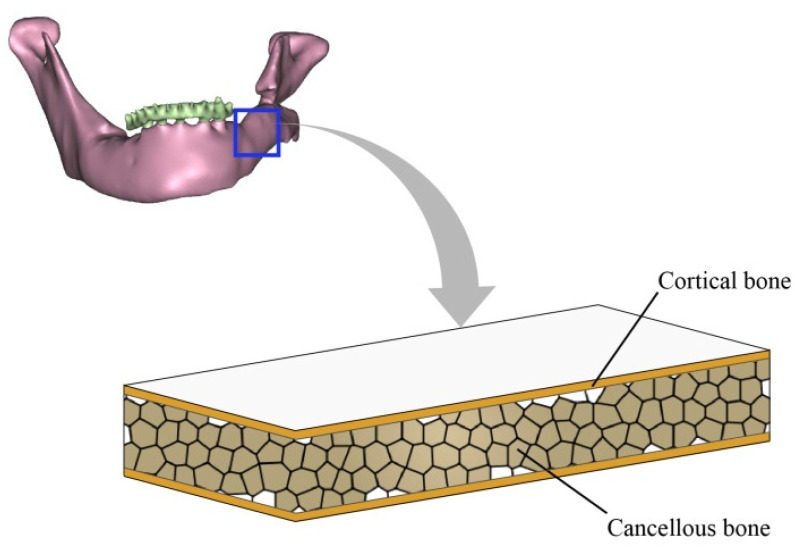
Laminated plate model of human mandibular bone.

The cancellous bone in the mandible was assumed to be elastically isotropic in this analysis, with an elastic modulus of 1.37 GPa and a Poisson ratio of 0.3 [30]. According to its anatomic structure, the mandible is represented by a laminated plate comprising three layers [31], as shown in Figure 2. The effective elastic properties were calculated through classical laminate theory (e.g., [32]):

(1) $$\left\{\begin{array}{l} E_{x_{0}}=\frac{A_{11}A_{22}-A_{12}^{2}}{A_{22}H}\\ E_{y_{0}}=\frac{A_{11}A_{22}-A_{12}^{2}}{A_{11}H}\\ G_{x_{0}y_{0}}=\frac{A_{66}}{H}\\ v_{x_{0}y_{0}}=\frac{A_{12}}{A_{22}} \end{array}\right.$$

where H is the thickness of the bone; $A_{ij}$ are the components of the stiffness matrix, defined as

(2) $$A_{ij}=\sum_{k=1}^{3}{\left({\bar{C}}_{ij}\right)}_{k}t_{k}$$

in which ${\left({\bar{C}}_{ij}\right)}_{k}$ are the stiffness matrix components of the 𝑘th layer and $t_{k}$ is the thickness of the 𝑘th layer.

### 2.2. Stress Analysis Based on Complex Stress Functions

The stress distribution in an orthotropic plate with elliptic or circular openings has been extensively explored in the literature. Analytical solutions of the stress distribution in orthotropic homogeneous plates with circular openings that are filled by an elastic material with different elastic properties have been derived by Lekhnitskii [33]. As has been stipulated, when the opening (far from the edge) is relatively small in comparison with the plate size, the plate could be simplified as infinite, and the effect of the external edge is neglected. The general solutions of the complex stress functions and stress components in this analysis followed those in [18,33]. Only the formulas are reproduced and explained here with consistent notation, given by Equations (3)–(9).

#### 2.2.1. General Solution of Stress Functions

**Figure 3:**
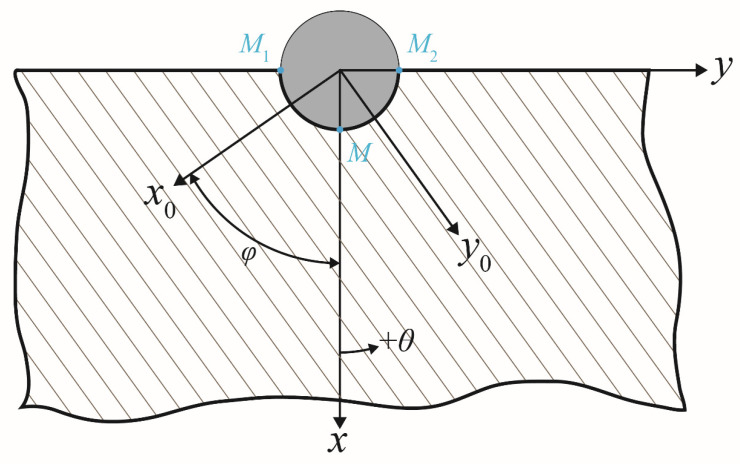
Geometry of the screw and bone and coordinate systems for analysis.

A Cartesian coordinate system x-y was set up, such that the displacement of the pin was in the direction of the positive x-axis, as shown in Figure 3. The radii of the screw and screw hole are identical and are denoted by r. The resultant force P from the pin to the plate is known. The principal material directions of the plate are such that the intersection angle between the principal material direction $x_{0}$ and coordinate axis x is φ. The Young’s moduli along the principal material directions $x_{0}$ and $y_{0}$ are $E_{x_{0}}$ and $E_{y_{0}}$, respectively. The shear modulus is $G_{x_{0}y_{0}}$, and the Poisson ratio characterizing the lateral shrinkage in direction $y_{0}$ caused by extension in direction $x_{0}$ is $v_{x_{0}y_{0}}$. Plane stresses were derived by solving the governing biharmonic differential equation in the complex domain [33]:

(3) $$\left\{\begin{array}{l} \sigma_{x}=2Re[\mu_{1}^{2}\phi_{1}^{\prime }\left(z_{1}\right)+\mu_{2}^{2}\phi_{2}^{\prime }\left(z_{2}\right)]\\ \sigma_{y}=2Re[\phi_{1}^{\prime }\left(z_{1}\right)+\phi_{2}^{\prime }\left(z_{2}\right)]\\ \tau_{xy}=-2Re\left[\mu_{1}\phi_{1}^{\prime }\left(z_{1}\right)+\mu_{2}\phi_{2}^{\prime }\left(z_{2}\right)\right] \end{array}\right.$$

where $\phi_{k}$ are complex stress functions and the first-order derivatives of functions $\phi_{k}$ are with respect to $z_{k}=x+\mu_{k}y$. The variables $\mu_{k}$ are the complex roots of the characteristic equation in the coordinate system x-y, while $\mu_{k_{0}}$ for principal coordinate system $x_{0}$-$y_{0}$. According to Lekhnitskii [33], if the resultant force components are known and displacements $u^{*}$, $v^{*}$ at the plate hole are given by

(4) $$\left\{\begin{array}{c} u^{*}=\alpha_{0}+\sum_{m=1}^{\infty }\left(\alpha_{m}e^{im\theta }+{\bar{\alpha }}_{m}e^{-im\theta }\right)\\ v^{*}=\beta_{0}+\sum_{m=1}^{\infty }\left(\beta_{m}e^{im\theta }+{\bar{\beta }}_{m}e^{-im\theta }\right) \end{array}\right.$$

The general solution of stress functions $\phi_{1}$ and $\phi_{2}$ could be expressed as

(5) $$\left\{\begin{array}{l} \phi_{1}\left(z_{1}\right)=A\;ln\xi_{1}+\frac{2\left({\bar{\alpha }}_{1}q_{2}-{\bar{\beta }}_{1}p_{2}\right)+\omega r\left(iq_{2}+p_{2}\right)}{2D\xi_{1}}+\sum_{m=2}^{\infty }\frac{{\bar{\alpha }}_{m}q_{2}-{\bar{\beta }}_{m}p_{2}}{{D\xi }_{1}^{m}}\\ \phi_{2}\left(z_{2}\right)=B\;ln\xi_{2}-\frac{2\left({\bar{\alpha }}_{1}q_{1}-{\bar{\beta }}_{1}p_{1}\right)+\omega r\left(iq_{1}+p_{1}\right)}{2D\xi_{2}}-\sum_{m=2}^{\infty }\frac{{\bar{\alpha }}_{m}q_{1}-{\bar{\beta }}_{m}p_{1}}{{D\xi }_{2}^{m}} \end{array}\right.$$

Here, the arbitrary constants were neglected since only derivatives of $\phi_{1}(z_{1})$ and $\phi_{2}(z_{2})$ were of interest for stresses; i is the imaginary unit, and θ is the angle with the positive x-direction, as shown in Figure 3; the bar sign “¯” denotes the complex conjugate; $\xi_{1}$ and $\xi_{2}$ are functions of complex variables $z_{1}$ and $z_{2}$, respectively; A, B, D, $p_{1}$, $p_{2}$, $q_{1}$, and $q_{2}$ are constants determined by the material properties. All aforementioned parameters are defined in [18,33].

#### 2.2.2. Boundary Conditions

The unique solutions of the stress components for the pin-loaded plate problem depend plainly on the unknown parameters $\alpha_{m}$ and $\beta_{m}$, which should fulfill specific boundary conditions of the problem. In the present work, we made use of the boundary conditions proposed previously by other researchers. According to Zhang and Ueng, the major displacement boundary conditions of this problem may be formulated as [18]

(6) $$\left\{\begin{array}{l} u_{1}=\frac{u_{0}}{c_{1}},\;v=0\;when\;\theta =\frac{\pi }{2}\\ u_{2}=\frac{u_{0}}{c_{2}},\;v=0\;when\;\theta =-\frac{\pi }{2}\\ \left(u_{0}-u\right)\cos\theta =v\sin\theta \;when\;-\frac{\pi }{2}<\theta <\frac{\pi }{2} \end{array}\right.$$

where u and v are the displacements of the points on the plate hole circumference in the x- and y-axis. $u_{0}$, $u_{0}$/$c_{1}$, and $u_{0}$/$c_{2}$ are the displacements of points M, $M_{1}$, and $M_{2}$, shown in Figure 3, respectively. Furthermore, denoting the friction coefficient as f, certain stress boundary conditions on the plate-hole circumference are also introduced [18]:

(7) $$\left\{\begin{array}{l} \tau_{r\theta }=0,\;\sigma_{rr}=0\;at\;\theta =\pm \frac{\pi }{2}\\ \int_{-\frac{\pi }{2}}^{0}\tau_{r\theta }rd\theta -\int_{0}^{\frac{\pi }{2}}\tau_{r\theta }rd\theta =f\left[\int_{-\frac{\pi }{2}}^{0}\sigma_{rr}rd\theta +\int_{0}^{\frac{\pi }{2}}\sigma_{rr}rd\theta \right] \end{array}\right.$$

#### 2.2.3. Exact Solution of Stresses

To determine the unknowns $\alpha_{m}$ and $\beta_{m}$, the expressions for displacements u and v are represented in the following form [18]:

(8) $$\left\{\begin{array}{l} u=-\frac{iU_{1}}{2}e^{i\theta }+\frac{iU_{1}}{2}e^{-i\theta }+\frac{U_{2}}{2}e^{2i\theta }+\frac{U_{2}}{2}e^{-2i\theta }+\frac{U_{3}}{2}e^{4i\theta }+\frac{U_{3}}{2}e^{-4i\theta }\\ v=\frac{V_{1}}{2}e^{i\theta }+\frac{V_{1}}{2}e^{-i\theta }-\frac{{iV}_{2}}{2}e^{2i\theta }+\frac{iV_{2}}{2}e^{-2i\theta }-\frac{iV_{3}}{2}e^{4i\theta }+\frac{iV_{3}}{2}e^{-4i\theta } \end{array}\right.$$

where $U_{i}$ and $V_{i}$ are unknown constants. Substituting the expressions for u and v into Equation (6), the expressions of $U_{i}$ and $V_{i}$ in terms of $u_{0}$, $c_{1}$, and $c_{2}$ are obtained. Comparing the corresponding terms from Equations (4) and (8), the unknowns $\alpha_{m}$ and $\beta_{m}$ can then be found. Thus, the stress components $\sigma_{xx}$, $\sigma_{yy}$, and $\tau_{xy}$ can be obtained from Equations (3) and (5). Applying the stress transformation between the polar and Cartesian coordinate systems, compact solutions for stress components in the polar coordinate system can be expressed as [18]

(9) $$\left\{\begin{array}{l} \sigma_{rr}=\frac{1}{\pi }\frac{P_{x}}{Sr}\sum_{k=1}^{3}\left(C_{k}^{\sigma }\cos(2k+1)\theta +S_{k}^{\sigma }\sin(2k+1)\theta \right)\\ \tau_{r\theta }=\frac{1}{\pi }\frac{P_{x}}{Sr}\sum_{k=1}^{3}\left(C_{k}^{\tau }\cos(2k+1)\theta +S_{k}^{\tau }\sin(2k+1)\theta \right)\\ \sigma_{\theta \theta }=\frac{P_{x}}{2r}\left(\sigma_{\theta 1}+\sigma_{\theta 2}+\sigma_{\theta 3}+\sigma_{\theta 4}+\sigma_{\theta 5}\right) \end{array}\right.$$

where S, $C_{1}^{\sigma }$, $S_{1}^{\sigma }$, $C_{1}^{\tau }$, $S_{1}^{\tau }$, etc. are expressed in detail in Appendix A; $P_{x}=P\cos\theta_{1}$ is the x-component of screw-load P, in which $\theta_{1}$ is the angle between the screw-load direction and the positive x-direction. Unknowns $u_{0}$, $c_{1}$, and $c_{2}$ are determined by substituting the stress components into the stress boundary condition, e.g., Equation (7).

For a finite-width plate, the stress concentration factor can be calculated by the use of the finite-width correction factor [34,35], which is a scale factor defined as the stress concentration factor of an infinite- to a finite-width plate. Thus, the stress concentration of the finite-width plate can be calculated with the specification of the finite-width correction factor and the stress concentration of the infinite plate with a similar opening and material. Since only trends and relative contributions from the inherent parameters to stress concentration were in focus in this work, the finite-width corrections were not used here.

## 3. Results and Discussions

The stress distributions around the screw hole depend on the elastic properties of cortical and cancellous bone, the friction coefficient, and the loading direction. To illustrate the influence of different parameters, we determined the numerical values of the stresses for the different regions in the mandibular bones based on the analytical model. The friction coefficient between the bones and the porous titanium alloy was around 0.6 [36]. Thus, the friction coefficient f in the present work varied from 0, 0.2, 0.4, 0.6 to 1, where the frictionless case would correspond to an unbonded screw. With the increase of f, the screw is considered to become increasingly bonded to the bone material. For each friction coefficient value, the profiles of the radial normal stress $\sigma_{rr}$ and the circumferential shear stress $\tau_{r\theta }$ were calculated for different cross-angles φ, i.e., 0°, 15°, 30°, 45°, 60°, 75°, and 90°, between the loading direction and the direction of the main material axis.

The stress distributions around the screw holes in different regions on the mandible were obtained for different combinations of the friction coefficient and the cross-angle. The analytic model predicts the average stresses for the homogenized bone laminate. The dimensionless relative thickness $t_{r}$ of the cortical bone layer is defined as

(10) $$t_{r}=\frac{2t_{cort}}{2t_{cort}+t_{can}}$$

where $t_{cort}$ is the thickness of the cortical bone layer and $t_{can}$ is the thickness of the cancellous bone layer. The relative thickness varies for different regions of the mandible bone. For the sake of simplification, the relative thickness for each of the superficial cortical bone layers was assumed as 0.2, 0.4, 0.5, 0.6, 0.8, and 1.0 for the symphysis, body, angle, ramus, condyle, and coronoid bones, respectively. For a relative thickness of 1.0, the bone is comprised only of the stiff cortical bone, which can be assumed to be the case for the coronoid part of the mandible. The effective elastic property of the mandible bone is orthotropic due to the symmetry and the stiffness values calculated from classical laminate theory, as listed in Table 2.

### 3.1. Average Stresses over the Cross-Section of the Laminated Bone

**Figure 4:**
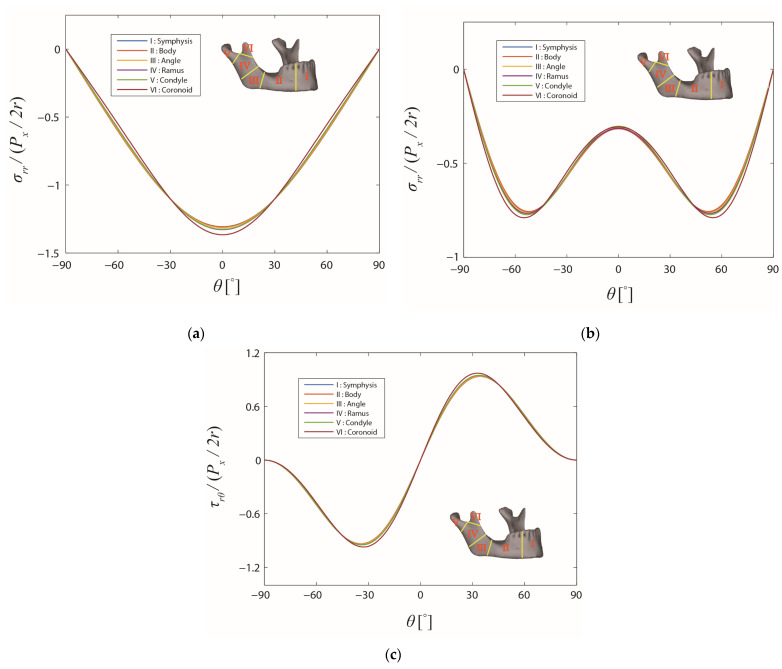
Stress distributions on the boundary of the screw hole in the different regions of the mandible. The friction coefficient f and the cross-angle φ are: (**a**) 0 and 0°, (**b**) 1 and 90°, and (**c**) 1 and 90°, respectively.

It was found that the differences between the stress distributions in different regions were not significant for the various values of f and φ. In order to limit the number of combinations of f and φ, only the distributions of the normalized stresses $\sigma_{rr}/(P_{x}/2r)$ for f = 0 and φ = 0° and $\tau_{r\theta }/P_{x}/2r$ for f = 1 and φ = 90°, which are two extreme cases, are illustrated in Figure 4. Shear stress $\tau_{r\theta }$ does not exist when the friction coefficient f = 0 and, thus, was not demonstrated. As depicted, different line colors imply the stresses in different regions, which are indicated by red roman numerals. It is clear from Figure 4 that, for the two extreme cases, the distributions of both the normalized radial and shear stresses were not distinguishable for different regions of the mandible, except for the coronoid region. Notably, the computational method was deemed to be only valid under the assumption that the screw was located far from the bone edge, which means that the stress distributions in the condyle and coronoid regions were less reliable. When there was no friction, the maximum radial stress occurred at the location along the loading direction (Figure 4a), as expected. With the presence of friction, the shear stress increased (Figure 4c), and the distribution of the radial stress varied with its maximum location moving away from the loading direction, as can be seen in Figure 4b.

**Figure 5:**
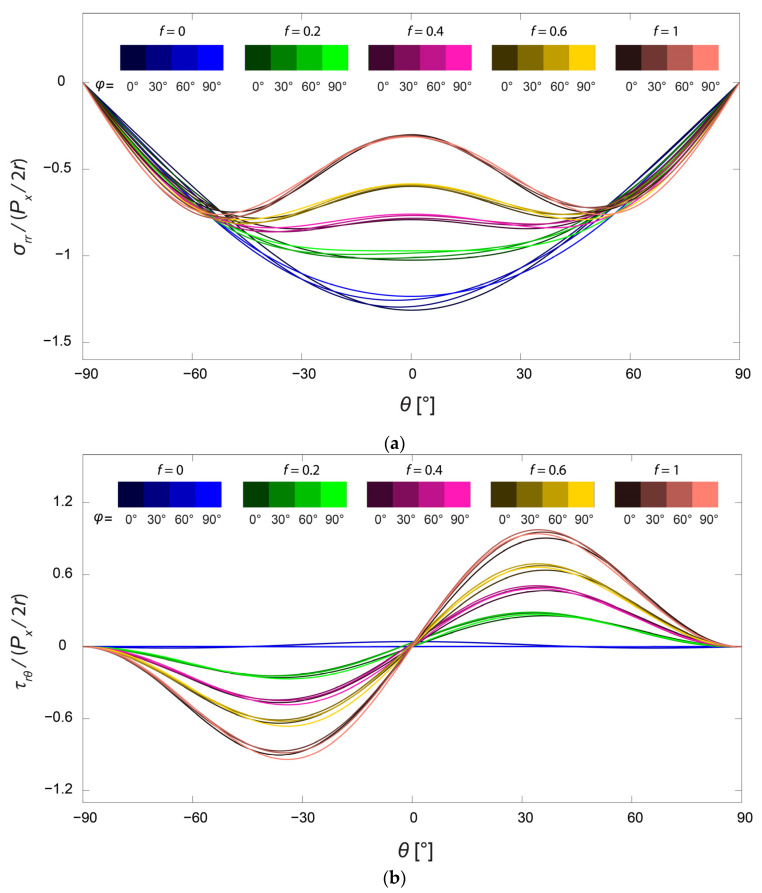
Distributions of normalized (**a**) radial stress and (**b**) shear stresses on the boundary of the screw hole in the angle region of the mandible. The friction coefficient f takes values from 0, 0.2, 0.4, 0.6, and 1, and the cross-angle φ takes values from 0°, 30°, 60°, and 90°.

Since the differences of the average stress distributions over the mandible were fairly small, the angle region in the mandible, where the largest percentage of mandibular fractures occur [37], was chosen to study the influence of the friction coefficient f and the cross-angle φ in more detail. In Figure 5, the stress distributions are demonstrated for the angle region (denoted as III), where different colors are representations for different values of the friction coefficient f and different brightness intensities stand for different values of the cross-angle φ. As can be observed, the stress distribution curves could be divided approximately into five groups with different levels of friction, for both the normalized radial and shear stresses. With the increase of the friction coefficient, the radial stress level around the screw hole gradually became lower [18,22]. Simultaneously, the distribution for the radial stress in radial direction $\sigma_{rr}$ changed with the high-stress region spreading out bilaterally from θ = 0°. Comparing the curves in each group, the effect of the loading direction, φ, can be observed only under closer scrutiny, and the stress distributions remained relatively constant. The variation of the friction coefficient, f, obviously had a larger impact on the stress distributions [38]. However, readers should bear in mind that the stress distributions derived in this work are only valid for screw–bone contact with large values of the friction coefficient, i.e., the bond between the screw and the bone should be tight.

**Figure 6:**
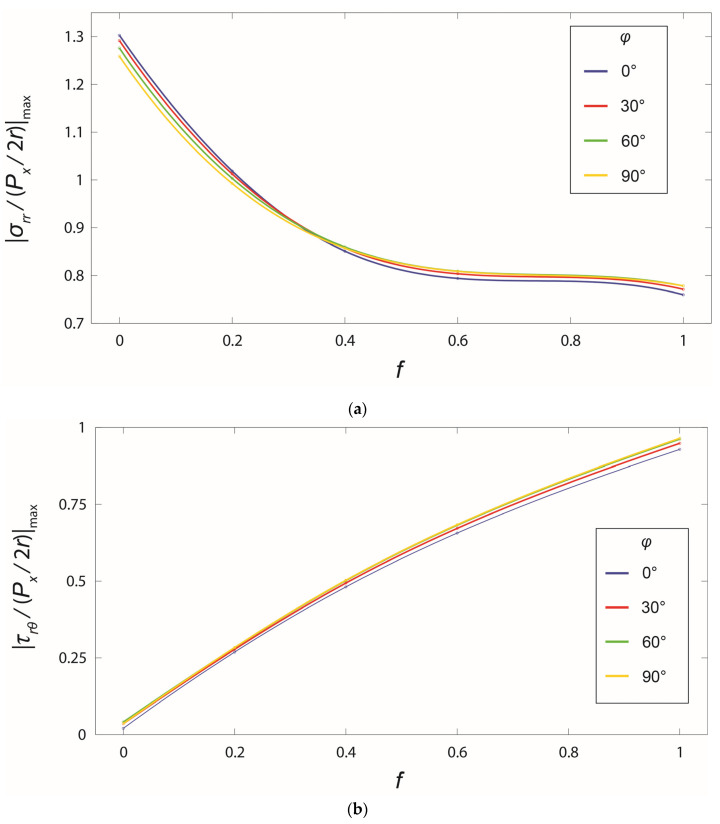
Change of maximum normalized stresses in the angle region with respect to the friction coefficient f and the cross-angle φ : (**a**) radial stress and (**b**) shear stress.

The critical point for possible damage formation, e.g., cracking, is at the location of the maximum stress levels. To demonstrate the influence of the friction and loading direction, the curves of the maximum stresses for the angle region with respect to f and φ are plotted in Figure 6. With the increase of the friction coefficient, the maximum radial stress decreased while the maximum shear stress increased steadily. On the contrary, the maximum stresses varied only within a small range when the loading direction varied. Not only for the stress distributions in Figure 5, but also for the maximum stresses, the friction played a more important role than the direction of the load.

### 3.2. Potential Applications of the Analytical Model

**Figure 7:**
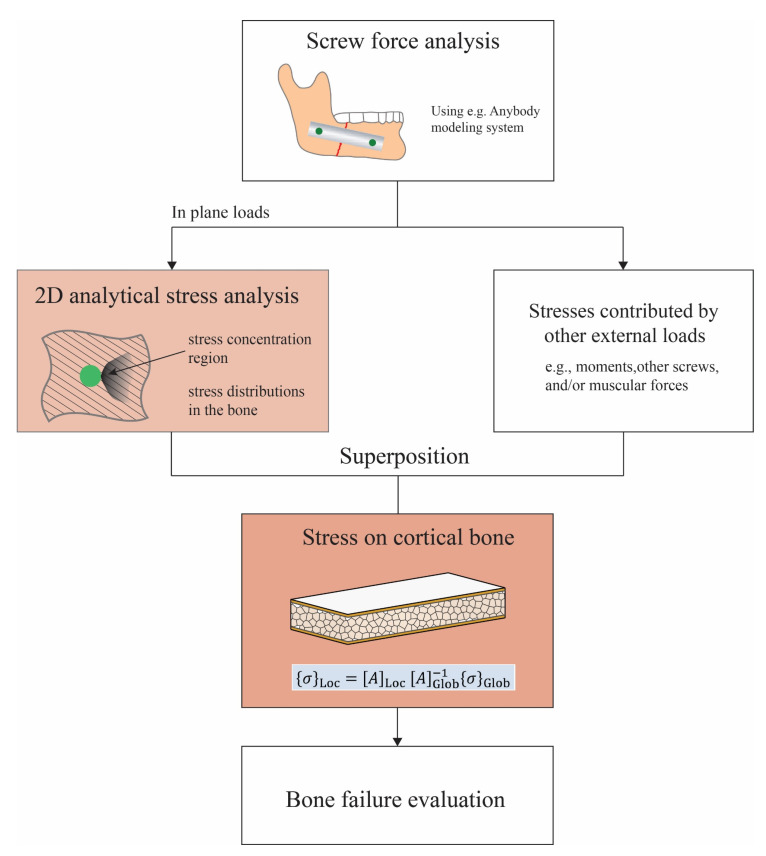
Schematic of a more-comprehensive approach to the prediction of bone failure from mandible implant screws.

It is important to keep in mind that the current analytical method could be used together with other analysis methods to implement a complete analysis of failure prediction in the case of in-plane loading. A schematic procedure is proposed and shown in Figure 7. The present study was limited to the highlighted orange box in Figure 7, i.e., the two-dimensional stress analysis under in-plane loading. The schematic shows how this analysis could fit into a larger framework. As can first be seen, the kinetics information of the mandible and the implant could be obtained through a macro-level analysis using commercial software, e.g., in [39]. The in-plane loads applied to the local screws were then used as the input for the 2D analytical stress analysis. The overall stress distributions around the screw hole were determined by superposing the contributions from the in-plane loads and other loads, e.g., moments and transverse loads from the muscular forces, which most likely should be treated numerically. Eventually, the stress distributions will be further evaluated, using currently available bone fracture criteria such as the Rankine criterion, the Mohr–Coulomb criterion, and the Tsai–Wu criterion [40], to predict bone failure.

According to different fracture criteria, it is preferable that the stress states in each bone layer are known, since the failure conditions will be different for different bone structures. The average stresses over the thickness of the laminated bone were calculated in Section 3.1. The local stresses in each layer, e.g., the cortical bone layer, are determined and analyzed hereafter. With only in-plane deformation considered, the average strain vector $\varepsilon_{lam}$ of the bone laminate analogy (cf. Figure 2) is expressed as

(11) $$\varepsilon_{lam}=HA^{-1}\sigma_{lam}$$

where H is the thickness of the bone, $A^{-1}$ is the inverse of stiffness matrix A, and $\sigma_{lam}$ is the average stress vector of the laminated bone in a Cartesian coordinate system. Then, the constitutive equation of the cortical bone was used to calculate the local stresses in the cortical bone layer from the average strains:

(12) $$\sigma_{cort}=D_{cort}A^{-1}\sigma_{lam}$$

in which $D_{cort}$ is the stiffness matrix of the cortical bone material under the plane stress state in the x-y coordinate system.

**Figure 8:**
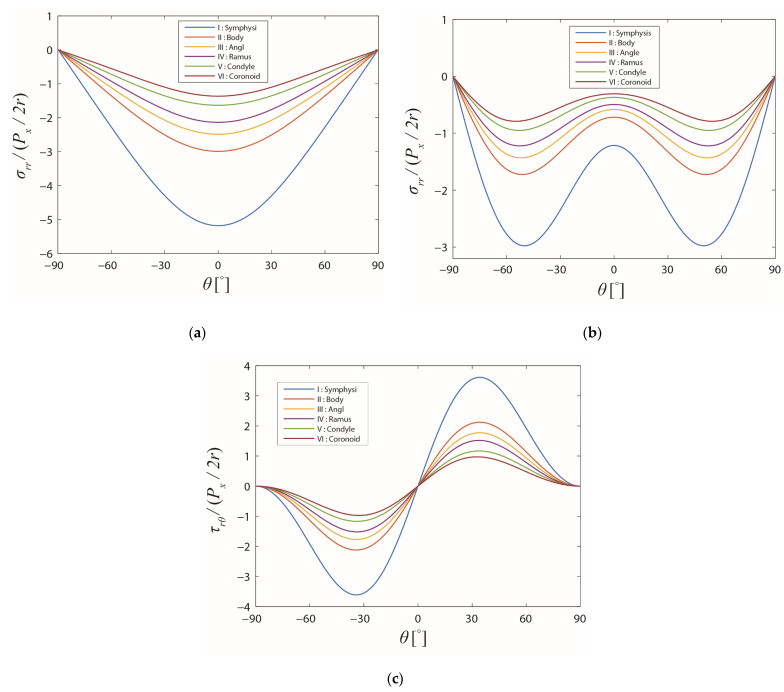
Stress distributions on the boundary of the screw hole in the different regions of the mandible in the cortical bone layer. The friction coefficient f and the cross-angle φ are: (**a**) 0 and 0°, (**b**) 1 and 90°, and (**c**) 1 and 90°, respectively.

The stress profiles around the screw hole in the cortical bone layer are demonstrated in Figure 8. As can be seen in the diagram, the stress distributions in the cortical bone layer were similar to those for the average stresses over the entire cross-section. Nevertheless, the differences between the stresses in different regions of the mandible were more distinguishable. Applying the same in-plane force to the screw, the stress levels in the cortical bone of various regions were different depending on the relative composition of the cortical and cancellous bone. The largest maximum stress level was observed in the symphysis region and decreased with the screw location moving towards the coronoid region. If the same stress-based failure criterion was applied for the cortical bone, failure was more likely towards the symphysis region since the stresses were higher there.

## 4. Conclusions

The effects of the friction coefficient and the loading direction, on the stress distributions around the screw hole in the mandible, were investigated analytically in two dimensions. The loading direction with respect to the material axis of the anisotropic bone turned out to have a negligible influence on the stress distributions. The parametric studies of friction suggested that the friction coefficient had a significant influence on both the radial and shear stress distributions and their maximal values. In crushing failure, the radial normal stresses were the dominating ones, and a high degree of bonding, manifested here by a high friction coefficient, is desirable to mitigate the stresses and increase the strength. The stress distributions in each bone layer were also obtained from the average stresses through classical laminate theory. It was concluded that the analytical method was much easier and more general compared with the finite element analysis, which consists of tedious work such as creating the geometry, meshing, simulating, etc., and it could allow for the parametric study of the design parameters given that a two-dimensional approach is viable. In this case, the present model may be implemented in a larger framework to predict failure based on a suitable criterion. The long-term goal is the quantitative design of improved implants from a mechanical perspective. However, one should keep in mind that the proposed analytical method is only for in-plane loading of flat parts on the bone, where the effects of the finite dimensions, bending moments, and out-of-plane loads were not accounted for.

## Figures and Tables

**Table 1:** Orthotropic elastic properties of cortical bone [28].

Positions in Mandible	Elastic Moduli (GPa)			Poisson Ratios			Shear Moduli (GPa)		
	$E_{1}$	$E_{2}$	$E_{3}$	$v_{12}$	$v_{23}$	$v_{13}$	$G_{12}$	$G_{23}$	$G_{13}$
Symphysis	20.49	16.35	12.09	0.34	0.22	0.43	6.91	4.83	5.32
Body	21.73	17.83	12.70	0.34	0.20	0.45	7.45	5.51	5.53
Angle	23.79	19.01	12.76	0.3	0.22	0.41	7.58	4.99	5.49
Ramus	24.61	18.36	12.97	0.28	0.23	0.38	7.41	5.01	5.39
Condyle	23.50	17.85	12.65	0.24	0.25	0.32	7.15	5.15	5.50
Coronoid	28.00	17.50	14.00	0.23	0.28	0.28	7.15	5.30	5.75

**Table 2:** Effective orthotropic elastic properties of different regions of the mandible.

Mandible	$E_{x_{0}}$ (GPa)	$E_{y_{0}}$ (GPa)	$G_{x_{0}y_{0}}$ (GPa)	$v_{x_{0}y_{0}}$
Symphysis	5.20	4.37	1.80	0.33
Body	9.51	7.95	3.30	0.34
Angle	12.58	10.20	4.06	0.30
Ramus	15.31	11.57	4.44	0.28
Condyle	19.08	14.56	5.83	0.24
Coronoid	28.00	17.50	7.15	0.23

## Data Availability

Not applicable.

## Appendix A

The expressions of the coefficients in the expressions of $\sigma_{rr}$ and $\tau_{r\theta }$ are provided as follows:

(A1) $$S=2A_{c}\left(k-v_{x_{0}y_{0}}-A_{\varphi }\right)-B_{c}(k-v_{x_{0}y_{0}}+A_{\varphi })$$

(A2) $$\left\{\begin{array}{l} C_{1}^{\sigma }=\frac{B_{c}}{2}\left(3D_{1}+A_{\varphi }+6k-6v_{x_{0}y_{0}}\right)-A_{c}(D_{1}-3A_{\varphi }+4k-4v_{x_{0}y_{0}})\\ C_{2}^{\sigma }=-\frac{B_{c}}{2}\left(D_{1}+{3A}_{\varphi }-6k+6v_{x_{0}y_{0}}\right)+A_{c}(A_{\varphi }+D_{1})\\ C_{3}^{\sigma }={-B}_{c}(A_{\varphi }+D_{1})\\ S_{1}^{\sigma }=-2A_{c}\left(k-v_{x_{0}y_{0}}-A_{\varphi }\right)\tan\theta_{1}+B_{c}\left(\left(k-v_{x_{0}y_{0}}+A_{\varphi }\right)\tan\theta_{1}+B_{1}\right)\\ S_{2}^{\sigma }=2B_{1}\left(B_{c}-A_{c}\right)\\ S_{3}^{\sigma }=2B_{1}B_{c} \end{array}\right.$$

(A3) $$\left\{\begin{array}{l} C_{1}^{\tau }=-2A_{c}\left(k-v_{x_{0}y_{0}}-A_{\varphi }\right)\tan\theta_{1}+B_{c}\left(\left(k-v_{x_{0}y_{0}}+A_{\varphi }\right)\tan\theta_{1}-B_{1}\right)\\ C_{2}^{\tau }=S_{2}^{\sigma }\\ C_{3}^{\tau }=S_{3}^{\sigma }\\ S_{1}^{\tau }={-A}_{c}\left(A_{\varphi }+D_{1}\right)+\frac{B_{c}}{2}\left(3D_{1}+2k-2v_{x_{0}y_{0}}-3A_{\varphi }\right)\\ S_{2}^{\tau }=\frac{B_{c}}{2}\left(-A_{\varphi }+5D_{1}+2k-2v_{x_{0}y_{0}}\right)-A_{c}(A_{\varphi }+D_{1})\\ S_{3}^{\tau }=C_{3}^{\sigma } \end{array}\right.$$

where the coefficients $A_{1}$, $B_{1}$, $C_{1}$, $D_{1}$, $A_{c}$, $B_{c}$, $A_{\varphi }$, and $\tan\theta_{1}$ and different terms in the expressions of $\sigma_{\theta \theta }$ are expressed in detail in [18].
